# Supplementary material for: Antibacterial Activity of Co(III) Complexes with Diamine Chelate Ligands against a Broad Spectrum of Bacteria with a DNA Interaction Mechanism
Source: Pharmaceutics. 2021 Jun 24;13(7):946. doi: 10.3390/pharmaceutics13070946 (PMC8309019; doi:10.3390/pharmaceutics13070946)
Supplement: Supplementary file 1 [file pharmaceutics-13-00946-s001.zip › pharmaceutics-1244649-SI.pdf]

pharmaceutics

MDPI

Supplementary Materials: Antibacterial Activity of Co(III) Complexes with Diamine Chelate Ligands against a Broad Spectrum of Bacteria with a DNA Interaction Mechanism

Katarzyna Turecka, Agnieszka Chylewska, Michał Rychłowski, Joanna Zakrzewska and Krzysztof Waleron

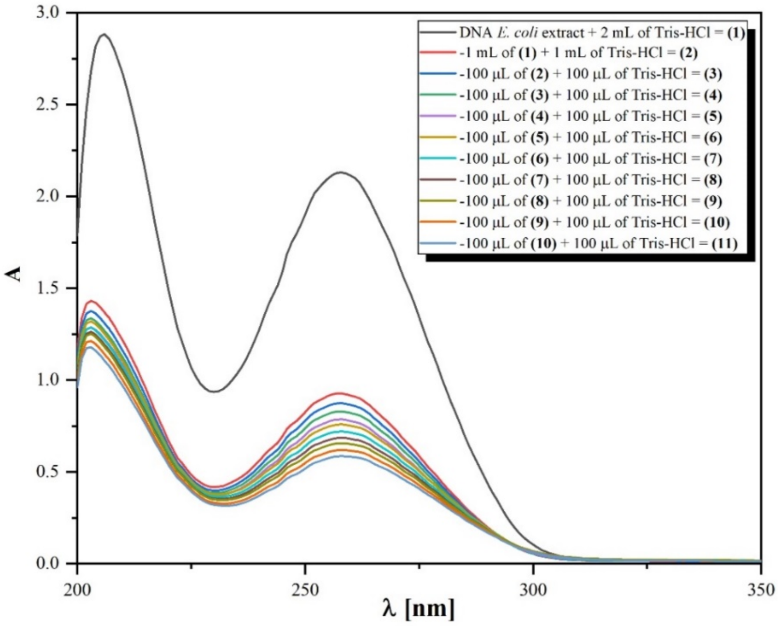

Figure S1. The Lambert-Beer law presentation used as a calibration curve for determining DNA concentration in Tris-HCl solutions studied.

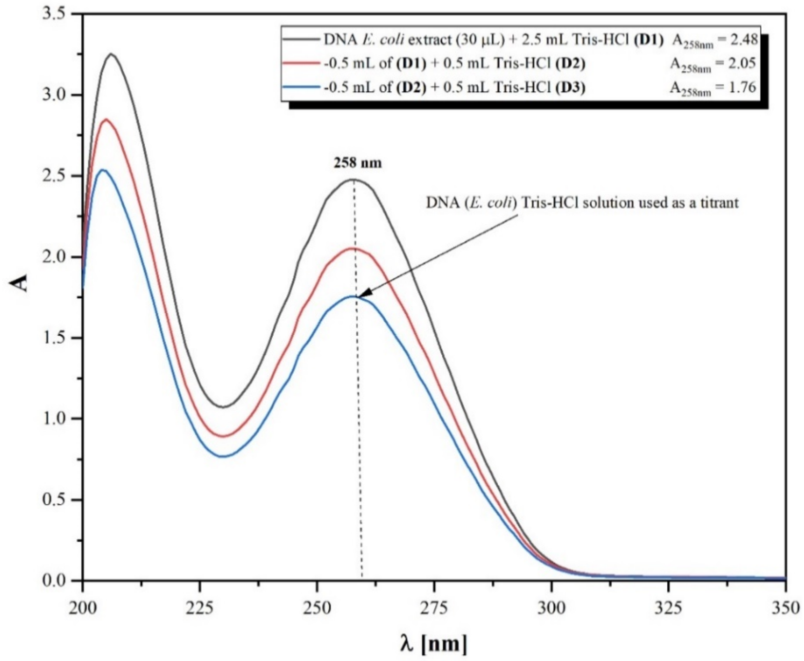

Figure S2. The procedure of selection the optimal concentration of DNA used further as a titrant during spectrophotometric titrations with complex **(2)**.

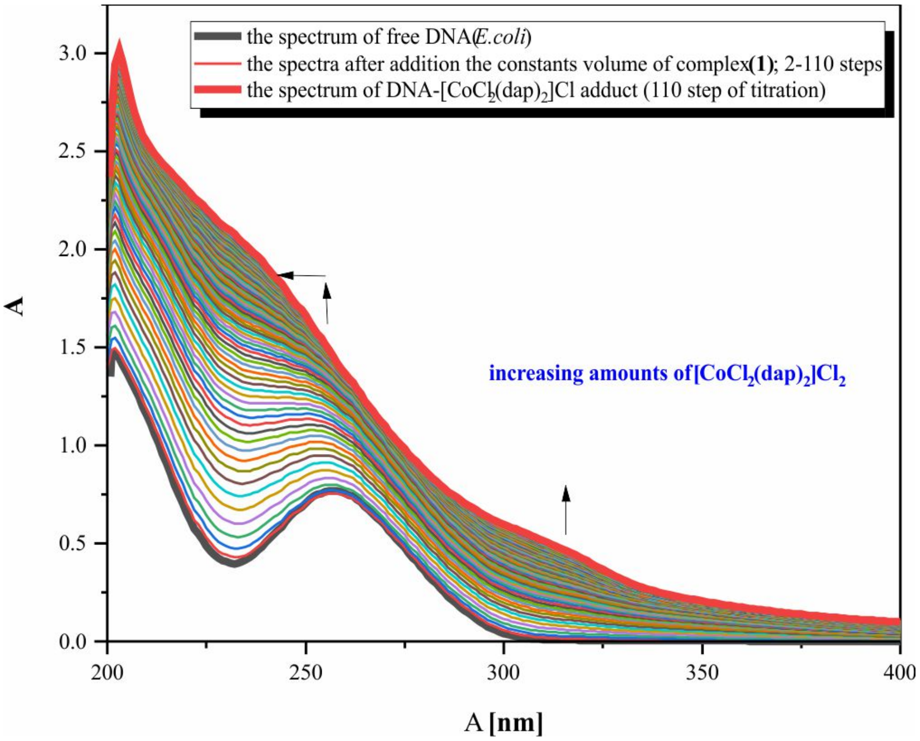

Figure S3. The spectrophotometric titration of DNA (*E. coli*) by using the complex **(1)** solution as a titrant.

[Co(dap)$_2$FLU]Cl$_2$ structure investigations

The [Co(dap)$_2$FLU]Cl$_2$ structure and the molar ratios, 1:2:1 for components Co^{III}:dap:FLU, respectively, were confirmed based on the data obtained by varied spectroscopy technique types, ^{1}H NMR (Figure S4), ATR (Figure S5) and UV-Vis (Figure S6) and were supported by elemental analysis results.

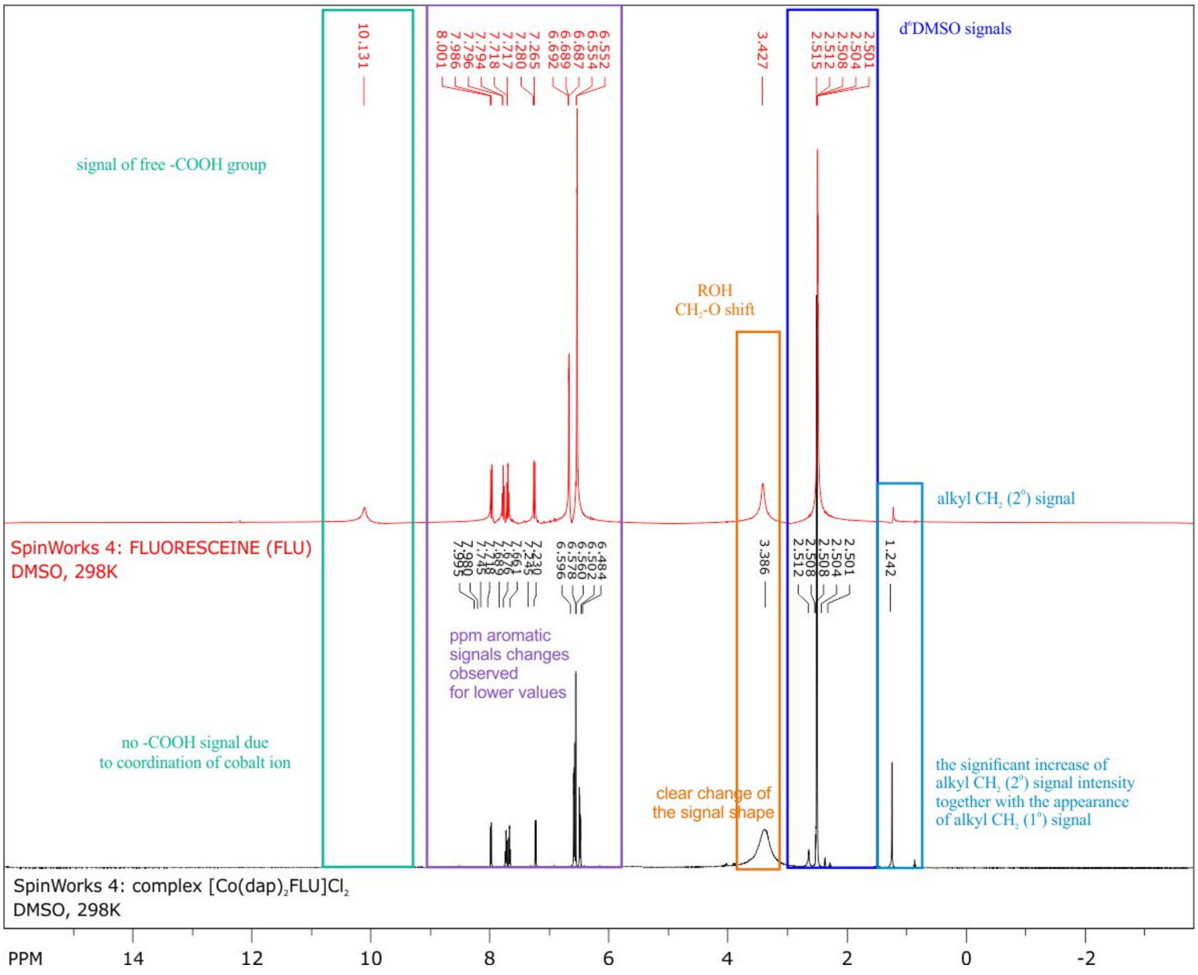

Figure S4. ^{1}H NMR spectra comparison together with signals assignments requested to confirm the $[Co(dap)_2FLU]Cl_2$ structure.

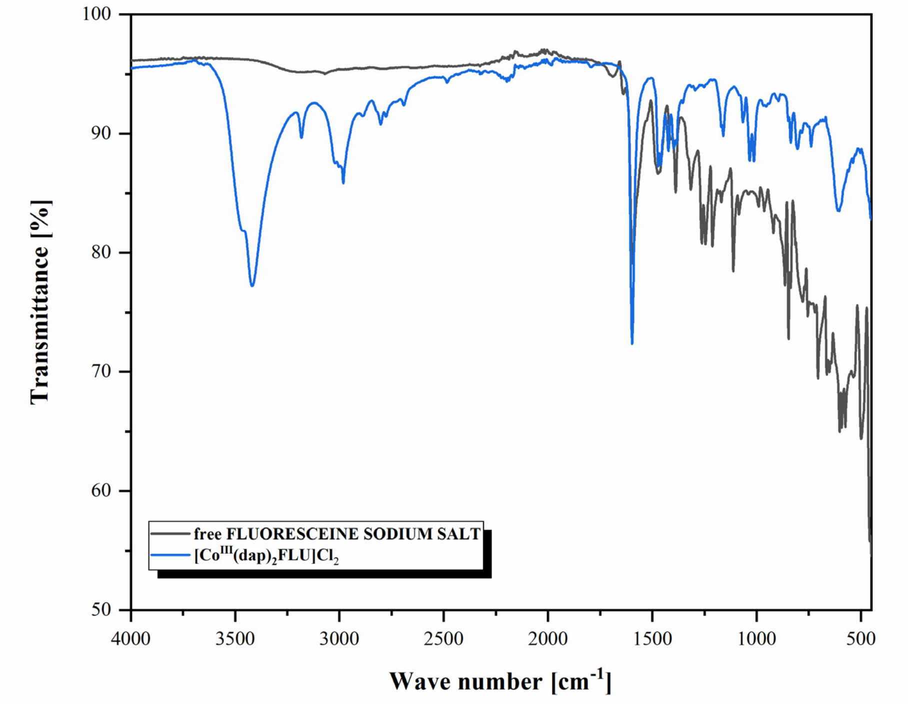

Figure S5. ATR spectra of Co(III) complex with neutral dap form (blue spectrum) and anionic form of FLU (fluoresceine) and free fluoresceine sodium salt (black spectrum).

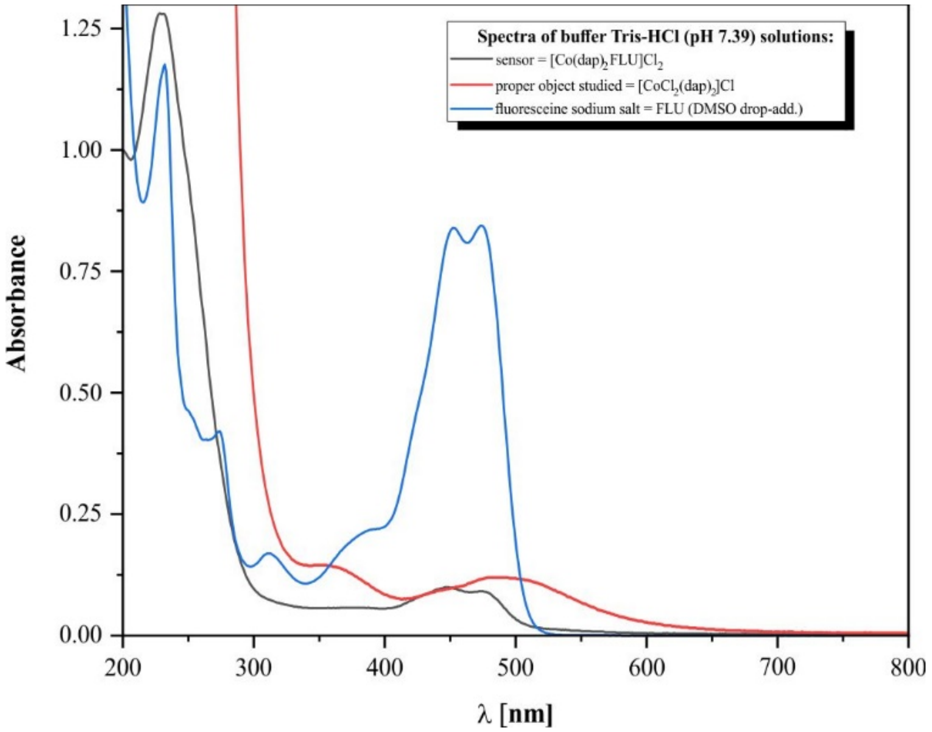

Figure S6. The comparison of electronic spectra obtained for buffer solutions: $[Co^{III}(dap)_2FLU]Cl_2$ sensor (black), fluoresceine sodium salt FLU (blue) and $[Co^{III}Cl_2(dap)_2]Cl$ (red); note the similarities and differences between absorption maxima positions in the compounds spectra above 400 nm.

Moreover, moles number of the choride ions (2) located outside the coordination sphere (as counter ions) was calculated based on potentiometric data by precipitation titrations with the chloride sensitive ion-selective electrode. The presence of Co(III) was proved by qualitative and quantitative tests. Both method procedures included the initial reduction of $[Co(dap)_2FLU]Cl_2$. Reduction related to cobalt oxidation state change from +III to +II. First instrumental test was carried out in an isoamyl alcohol medium with addition of ammonium thiocyanate solution. A blue color which appeared on the second day after the analysis (Figure S7) in the sample proves the presence of cobalt ion in the basic structural formula of the synthetic product.

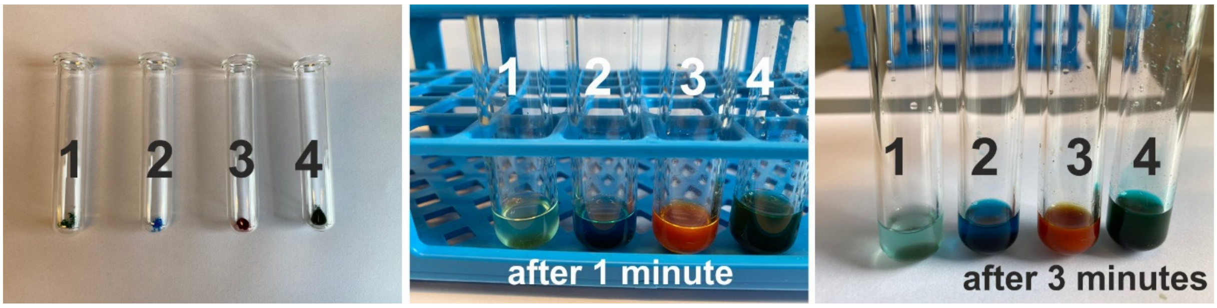

Figure S7. The analysis effect as a confirmation of the Co ions presence in the indicator formula: $[CoCl_2(en)_2]Cl$ (**1**); $[CoCl_2(dap)_2]Cl$ (**2**); FLU (**3**); $[Co(dap)_2FLU]Cl_2$ (**4**).

Interestingly, the trivalent cobalt ion was reduced in the preliminary step in the second type of assay, according to the standards. Indeed, the divalent cobalt ions were actually quantitatively characterised spectrophotometrically as an stoichiometric indicator of its trivalent precursor. The second method was carried out with aqueous solution samples and the methodology required to use the specific 1-nitroso-2-hydroxy-3,6-naphthalenedisulfonic acid disodium salt (Figure S8). The red colour flash light during shaking of solution identified the presence of the cobalt ions as one of the components.

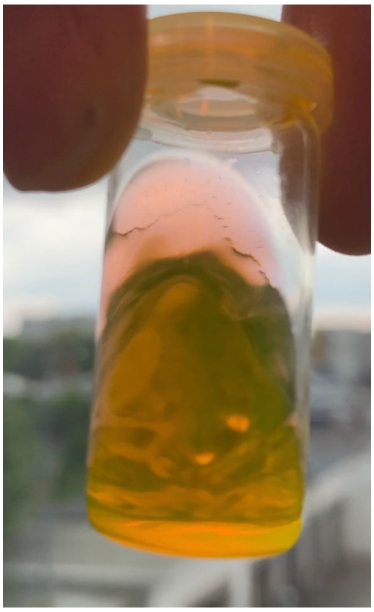

Figure S8. The effect of the qualitative analysis to confirm the Co(III) presence in the formula $[Co(dap)_2FLU]Cl_2$.

Based on the XRD experimental data (Figure S9), e.g. peak positions as well as signal intensities, crystallites (grain) sizes were calculated using Scherrer equation (eq. 1).

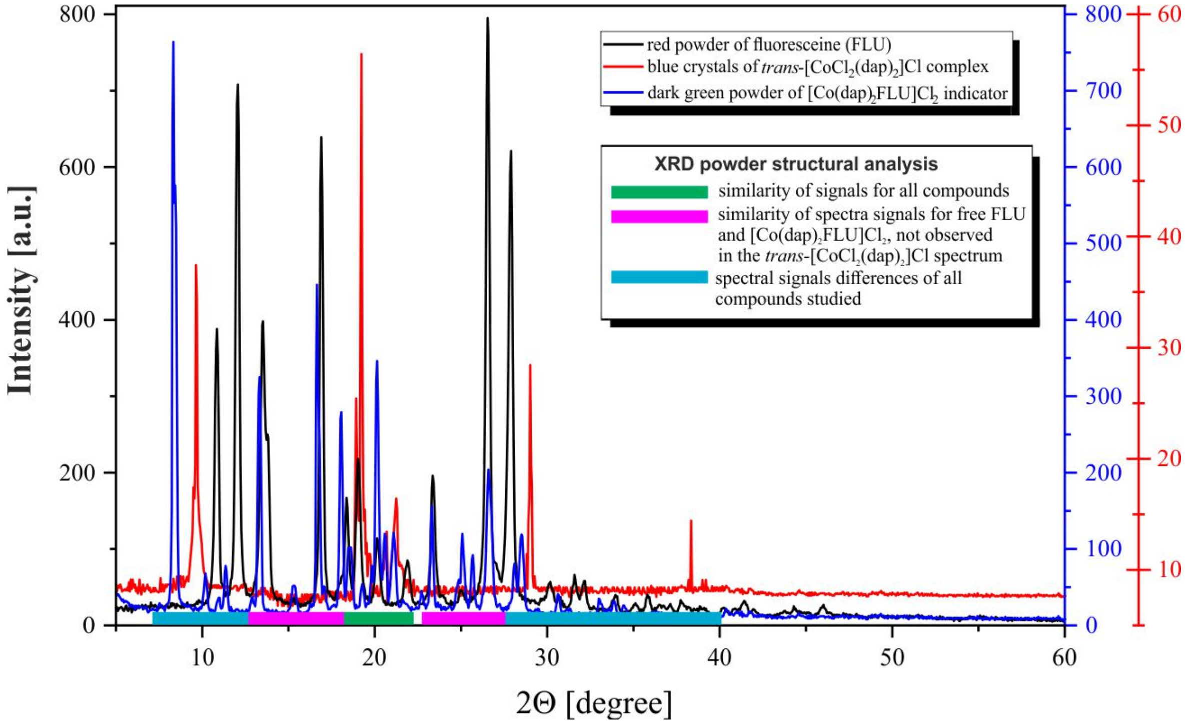

Figure S9. The XRD structural analysis prepared for solid states FLU and *trans*-$[CoCl_2(dap)_2]Cl$ solid states together with $[Co(dap)_2FLU]Cl_2$ designed intentionally as sensor to investigate cell penetration by the motif Co(III) coordinated by two 1,3-diaminepropane ligands as well as it localization inside the fungus cell.

$$D = \frac{K\lambda}{\beta\cos\theta}, \quad (1)$$

where:
D—crystallites size [nm]
K—0.9 (Scherrer constant)
λ—0.15406 nm (wavelength of the X-ray sources, Cu)

β—FWHM (radians)

θ—peak position (radians)

The detailed structural descriptors, calculated based on the XRD diffractometers data for solid state reagents used during synthesis of the $[Co(dap)_2FLU]Cl_2$ final product, were summarized and presented in Table S1.

Table 1. Crystallite sizes obtained by XRD powder method.

Signal No.	Peak position (2θ)	FWHM	Crystallite size [nm]	D (average)
**Fluoresceine sodium salt (FLU) (red powder of substrate)**				
**Signal No.**	**Peak position (2θ)**	**FWHM**	**Crystallite size [nm]**	**D (average)**
**1**	8.37775	0.25844	30.82172884	28 nm
**2**	13.31457	0.21499	37.20274511	
**3**	16.69176	0.23467	34.1536839	
**4**	18.04927	0.21221	37.90520757	
**5**	20.13662	0.24199	33.34247037	
**6**	26.63382	0.36792	22.1890795	
**7**	23.30736	0.20707	39.17272367	
**8**	28.41931	0.6090	13.45652541	
**9**	25.20596	1.06423	27.649125921	
***trans*-$[CoCl_2(dap)_2]Cl$ complex (blue crystals of substrate)**				
**Signal No.**	**Peak position (2θ)**	**FWHM**	**Crystallite size [nm]**	**D (average)**
**1**	9.39108	0.43298	18.40972268	18 nm
**2**	22.38715	0.41721	19.41071495	
**3**	21.27432	0.45012	17.95785988	
**4**	21.27432	0.43985	18.3771556	
**5**	26.16168	0.4899	16.64813483	
**$[Co(dap)_2FLU]Cl_2$ (dark green powder product)**				
**Signal No.**	**Peak position (2θ)**	**FWHM**	**Crystallite size [nm]**	**D (average)**
**1**	10.84171	0.28363	28.13517017	25 nm
**2**	12.04844	0.26385	30.27630855	
**3**	13.56928	0.53406	14.98017731	
**4**	16.9085	0.24056	33.3869447	
**5**	18.36573	0.3926	20.49777005	
**6**	19.04817	0.33467	24.06944899	
**7**	20.15996	0.35401	22.79267924	
**8**	23.37996	0.32776	24.75151811	
**9**	26.55406	0.27625	29.54737852	
**10**	27.87295	0.33096	24.73179501	

Although, the percentage elements contribution was determined (elemental analysis results), the coordination mode of FLU still needed to be improved. Due to above, additional techniques were performed and the structure of $[Co(dap)_2FLU]Cl_2$ was developed (Figure S10).

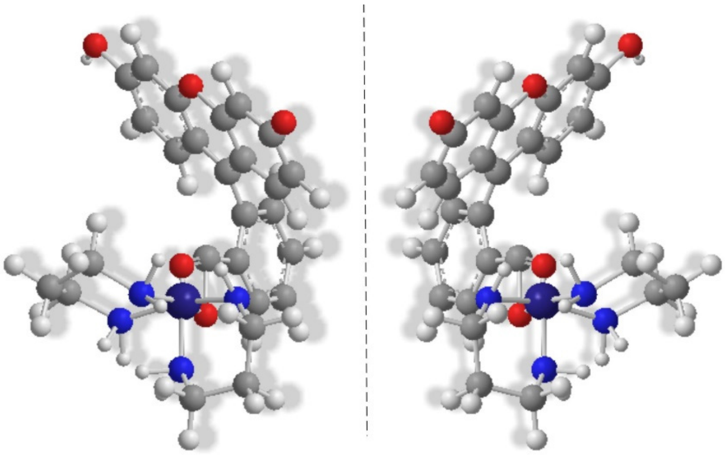

Figure S10. The proposed enantiomer structures of the coordination cation formula $[Co(dap)_2FLU]^{2+}$.

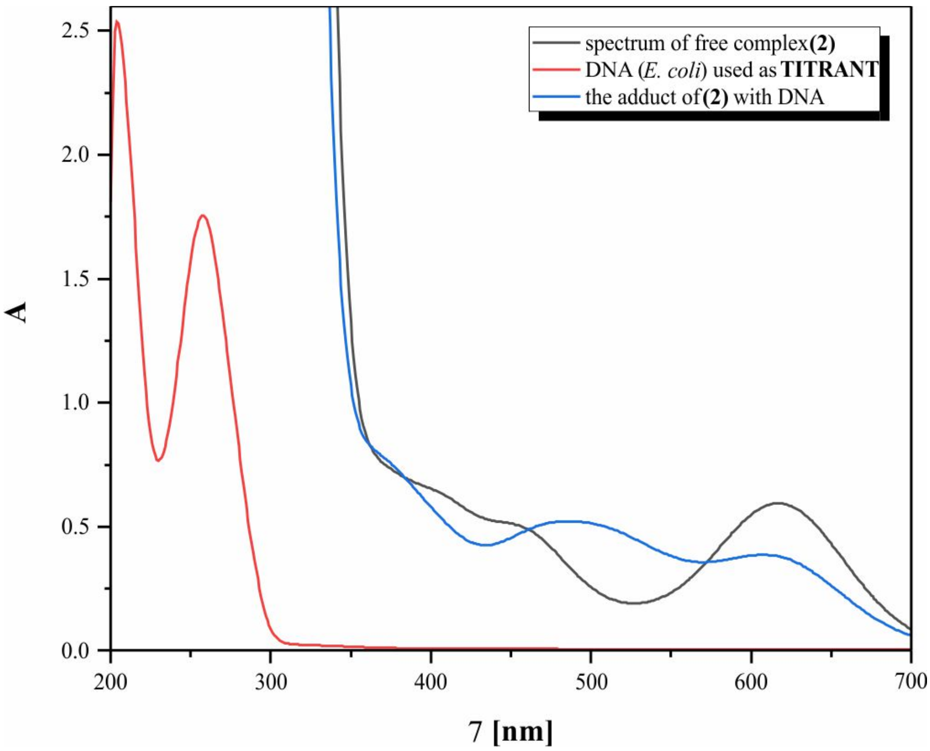

Figure S11. Absorption spectra of complex **(2)** in the presence of DNA (*E. coli*) : $c_{(1)}$ = 0.025 M; c_{DNA} = 2.68·10^{-4} M; the last spectrum of titration system presented the adduct formed between complex **(2)** and DNA.

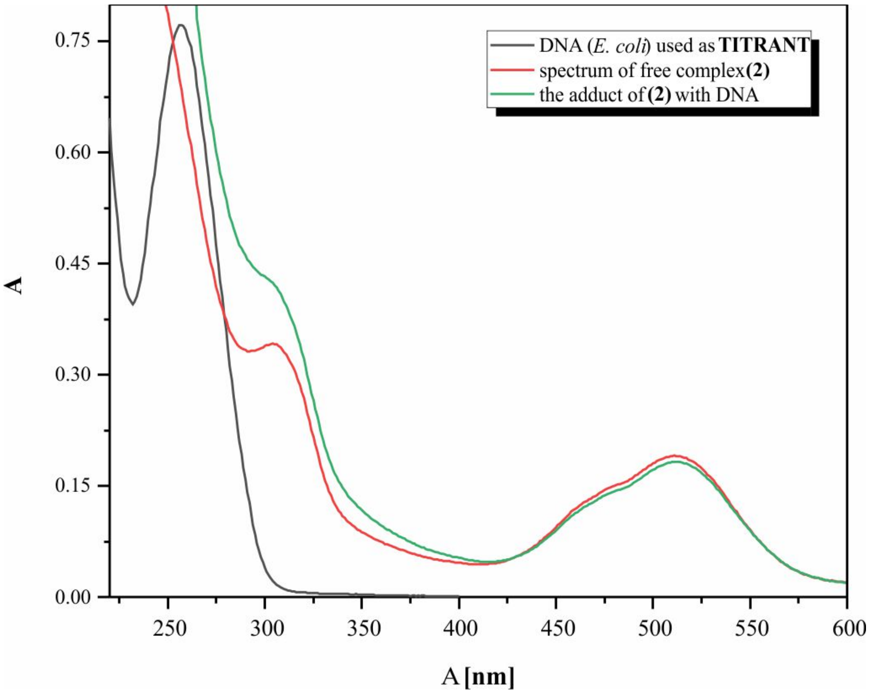

Figure S12. Absorption spectra of complex **(1)** in the presence of DNA (*E. coli*) : $c_{(1)}$ = 12 mM; c_{DNA} = 0.12 mM; the last spectrum of titration system presented the adduct formed between complex **(1)** and DNA.
